# The Correlation Between Parenting Styles and Self-Esteem of Medical Students: A Cross-Sectional Study

**DOI:** 10.1192/bjo.2022.168

**Published:** 2022-06-20

**Authors:** Fahad Gul, Khawar Abbas, Sajeel Saeed, Muhammad Arish, Tehseen Haider, Kashif Tousif, Jawad Basit

**Affiliations:** Rawalpindi Medical University, Rawalpindi, Pakistan

## Abstract

**Aims:**

Among various factors that determine an individual's self-esteem, parenting styles play a very significant role in this regard. Certain parenting styles that are practiced around the globe include authoritarian, authoritative and permissive parenting. The study aimed to investigate the co-relation between parenting styles and self-esteem among medical students.

**Methods:**

A cross-sectional study was conducted among the MBBS students of Rawalpindi Medical University, Rawalpindi from December 2020 to March 2021. 255 students took part in this study. An online survey was prepared by using Parental Authority Questionnaire-Short Version and Rosenberg Self-Esteem Scale and students were asked to fill it. Simple Random sampling technique was applied. SPSS version 26.0 was used to enter and analyze data. Pearson Correlation, Logistic regression and Hierarchal regression analysis were applied.

**Results:**

Out of 230, 60% of the sample population experienced authoritative, 29% experienced authoritarian whereas 11% experienced permissive style of parenting. Authoritative (r = 0.283, p < 0.001) and authoritarian (r = -0.227, p < 0.001) parenting styles were significantly correlated with self-esteem. Authoritarian parenting style (AOR = 2.20, 95% CI: 1.17, 4.14) was significantly associated with self-esteem of the participants compared to authoritative parenting style.

**Conclusion:**

Results indicated that authoritative parenting was only parenting style that correlates positively with self-esteem which suggest authoritative parenting is the optimum parenting style in Pakistani culture.

